# Spatial analysis of condyle position according to sagittal skeletal relationship, assessed by cone beam computed tomography

**DOI:** 10.1186/2196-1042-14-36

**Published:** 2013-10-18

**Authors:** Jessica M Arieta-Miranda, Manuel Silva-Valencia, Carlos Flores-Mir, Ney A Paredes-Sampen, Luis E Arriola-Guillen

**Affiliations:** Department of Orthodontics, Universidad Mayor de San Marcos (UNMSM), Lima, Peru; Department of Dentistry, University of Alberta, Edmonton, Canada

**Keywords:** Condyle, Temporomandibular joint, Computed tomography, CBCT

## Abstract

**Background:**

The study aims to compare the condylar position in patients with different anteroposterior sagittal skeletal relationships through a cone beam computed generated tomography (CBCT) imaging generated space analysis.

**Methods:**

This was a retrospective study of clinically justified, previously taken CBCT images of 45 subjects. Based on a proper sample calculation, three groups of 15 CBCT images each were made according to their ANB angle and facial pattern: class I (normo facial pattern) and class II and III (long facial pattern). The CBCT images were of adult patients between 18 and 35 years old, with full permanent dentition at maximum occlusal intercuspidation. Anatomical references previously used by Ricketts for the condyle position inside the glenoid fossae were measured digitally through the EzImplant software. Analysis of variance, Tukey's, Kruskal-Wallis, and Mann–Whitney *U* statistical tests were used.

**Results:**

The upper distance of the condyle to the glenoid fossa was smaller in the class II and class III compared with the class I group. The anterior distance of the condyle to the articular eminence showed significant differences when comparing the class I with the class II and class III groups. No statistically significant difference was noted in the posterior condylar distance between the groups. The angle of the eminence showed differences between the three groups, while the eminence height showed significant difference when comparing the class I with class III group.

**Conclusions:**

Spatial differences existed for the condylar position in relation to the glenoid fossa for skeletal class I, class II, and class III, but these spatial differences may not be clinically relevant.

## Background

The mandibular condyle as well as the other structures of the temporomandibular joint (TMJ) are important in sustaining good occlusion and a balanced stomatognathic system. There are several factors that could affect the TMJ morphology and position, such as age, sex, facial growth pattern, pathological/functional alterations, decreased or increased muscular activity, occlusal force, and dental occlusion changes [1–4]. As a result of these changes, there is a remodeling and reconfiguration of the TMJ surfaces as an adaptation response [5]. However, the amount of this remodeling will depend on the mechanical and functional conditions to which adjacent structures are faced [4, 5].

The TMJ can be evaluated by various radiologic imaging techniques such as panoramic radiography, TMJ radiography, both open- and closed-mouth transcranial projections, linear tomography, computed tomography, and magnetic resonance imaging. The use of conventional radiographs has inherent limitations such as structural superimpositions in two-dimensional imaging, particularly in the region of the petrous temporal bone, the mastoid process, and the articular eminence, which indeed limits an accurate view of the TMJ [6]. Currently, cone beam computed tomography (CBCT) is an auxiliary diagnostic element that may provide theoretical advantages over 2D imaging of the TMJ. CBCT has been shown to provide three-dimensional (3D) high resolution imaging that allows the qualification and quantification of facial bone tissues in approximately real dimensions (1:1 ratio) without significant magnification or distortion [7–9]. However, it has to be noted that CBCTs have failed so far to demonstrate clearly superior diagnostic capability compared to axially corrected tomography, at least for the presence of osteophytes and erosions [10].

Previously, different authors have studied the relationship between the condyle and the glenoid fossa, using various measurements and tools [1, 3, 4, 11–14]. Furthermore, variations of condylar position in the glenoid fossa have been correlated in patients with different malocclusions. Ricketts [15] did publish a key study in this area by evaluating condylar position and size, both at rest and in occlusion, through TMJ laminography. This was based on a study by Brader [16] who compared actual bone structures (*ex vivo* skull measurements) with laminographic images. The level of precision, with a correction of 0.5 mm, was determined and its use was recommended for longitudinal studies.

The sagittal relationship between the maxilla and mandible may influence other adjacent structures of the craniofacial system, such as the TMJ. Thus, the morphology and condyle-glenoid fossa relationship could be compromised, in significant sagittal discrepancies, due to tension or compression forces that the surrounding tissues exert on the TMJ. This indeed could favor a continuous adaptation through remodeling processes to functional changes in the surrounding tissues [12, 13]. A vertical facial pattern is a factor considered in the condylar-glenoid fossa relation because patients with a long vertical facial pattern exhibit greater divergence of the palatal and mandibular plane influencing condylar rotation, which can be displaced with respect to a group of medium vertical pattern control [14].

For the above reasons, it has been hypothesized that skeletal sagittal and vertical discrepancy is a factor influencing condylar position [12, 13]. However, this information has not been reported yet with data obtained through CBCT imaging. Therefore, the purpose of this study was to compare the CBCT-based spatial analysis of the TMJ condylar position as related to the anteroposterior skeletal relationship.

## Methods

This study was approved by the UNMSM Ethics Committee. Sample size was calculated considering a mean difference of 1 mm for any of the linear distances considered (obtained from a preliminary pilot study) and a standard deviation of 0.7 mm. With a one-sided significance level of 0.01 and a power of 80%, a minimum of 15 patients per skeletal group was required.

All the CBCT images were obtained from previously available diagnostic data from patients currently under orthodontic treatment. These CBCT images were not specifically taken for this study but were already taken through the request of the treating professional.

The sample was divided into three groups based on their sagittal skeletal relationship (class I, class II, and class III) (Table 1). Assignment was determined using Steiner's ANB angle according to the following degree of severity: class I (2° to 4°), class II (5° to 10°), and class III (−3° to −6°). The vertical facial pattern was determined by S-Go/N-Me measures where if this proportion was smaller than 0.59 it was considered an increased vertical facial pattern.

**Table 1:** **Study group classification by sex, age, ANB, and vertical pattern**

Anteroposterior skeletal relationship	Number	Sex		Age (years)			ANB (deg)			Vertical pattern (S-Go/N-Me)		
		M	F	Min	Max	Mean	Min	Max	Mean	Min	Max	Mean
Class I	15	10	5	18	31	25	3	4	3.3	0.61	0.63	0.62
Class II	15	10	5	18	34	26	6	10	6.9	0.56	0.58	0.57
Class III	15	10	5	18	29	24	−5	−3	−3.4	0.55	0.57	0.56

The selection inclusion/exclusion criteria included the following:

- ■ CBCT images of adult patients (between 18 and 35 years old) with full permanent dentition at maximum occlusal intercuspidation and with long vertical facial pattern for class II and III.
- ■ Patients whose CBCT images were taken during orthodontic treatment were excluded.
- ■ Patients with condylar hyperplasia, lip and/or cleft palate, or with abnormal craniofacial syndromes and facial asymmetry were also excluded.

The CBCT images were taken using a Picasso Master 3D equipment (Vatech, Hwaseong, South Korea; settings set at 8 mA, 90 Kpv), with the patient properly positioned and at maximum teeth intercuspidation. These DICOM images were processed with the EzImplant software (Vatech, Hwaseong, South Korea) with a flat panel of 25 cm × 20 cm, 30 cm × 30 cm, whose field of vision of 20 cm × 19 cm included the areas of interest with dimensions of 672 × 672 × 496 pixels (510 MB) and a resolution of 0.3 mm × 0.3 mm × 0.3 mm.

**Figure 1:**
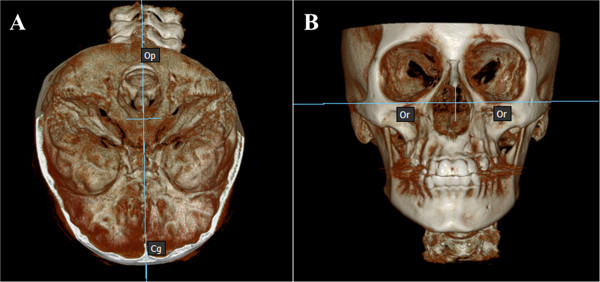
**Orientation of the skull in the Po-Or perpendicular to the sagittal Cg and Op. (A)** Axial view of the midsagittal plane. **(B)** Front view of the Frankfurt plane.

**Figure 2:**
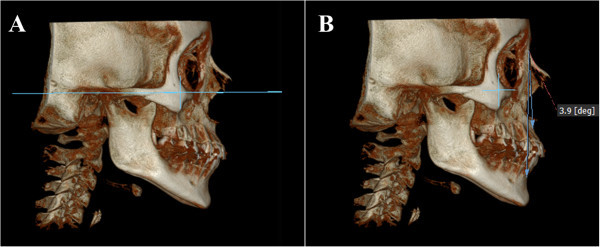
**Position of the skull to the right side. (A)** Sagittal view of the skull in the Frankfurt plane. **(B)** ANB angle and Steiner analyses to determine the skeletal pattern.

**Figure 3:**
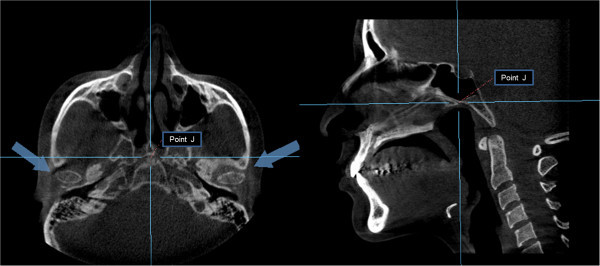
**Location of the condyles.** Position of the condyles in the axial plane using the point J (joint between the vomer and the sphenoid) (left). Confirmation of the point J in the sagittal view (right).

The patient's head scan was positioned based on the Frankfurt plane (Po-Or) perpendicular to the sagittal midline previously located in the axial view point opisthion (Op) and crista galli (Cg) (Figure 1); then the patient's head scan was turned to the right side in 3D view for the analysis of Steiner and to find the ANB angle (Figure 2). For class II and III skeletal relationship, the vertical facial pattern (S-Go/N-Me) was determined (Figure 3).

**Figure 4:**
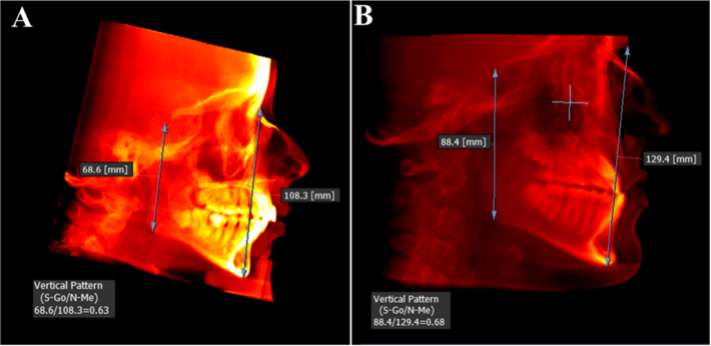
**Determination of vertical facial pattern (S-Go/N-Me). (A)** Vertical pattern in class II skeletal relationship. **(B)** Vertical pattern in skeletal class III relationship.

**Figure 5:**
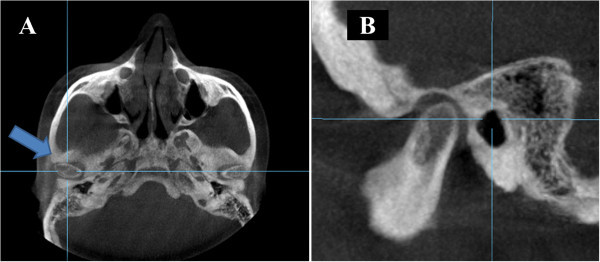
**Location of the rotary axes on the right condyle. (A)** Axial view of the right condyle. **(B)** Sagittal view of the right condyle.

**Figure 6:**
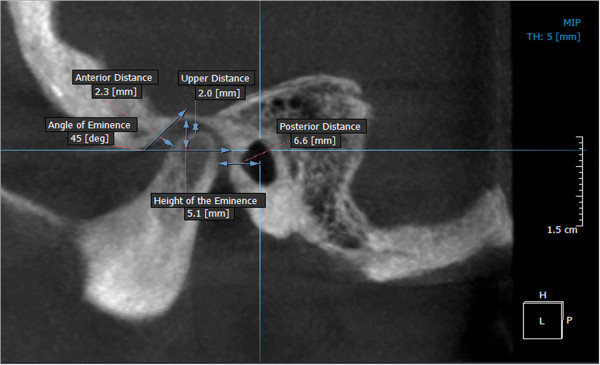
**Analysis in sagittal view of the right condyle.** At a thickness of 5 mm (MIP view) and 1.5-mm (zoom).

Thereafter, the axial view was used to locate the condyles, taking as reference the point ‘J’ (the joint between the vomer and the sphenoid), later confirmed in the sagittal view [17]. This reference point was then used to reproduce the imaging cuts at the same level for the subsequent measurements (Figure 4). The rotation axes were marked on the midpoint of the right condyle to obtain the sagittal cuts and proceed with the analysis of the condylar position (Figure 5). A thickness of 5 mm in the multiplane image (MIP) and a 1.5-mm zoom were used (Figure 6). The right condyle was selected to standardize measurements.

**Figure 7:**
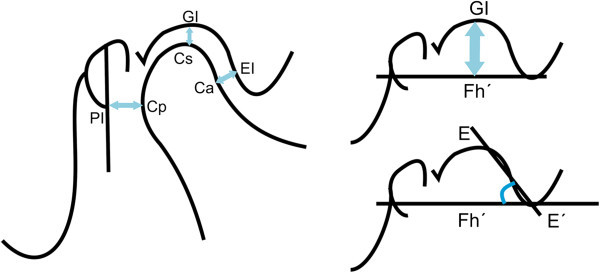
**Landmarks established for the analysis of condyle position.**

**Figure 8:**
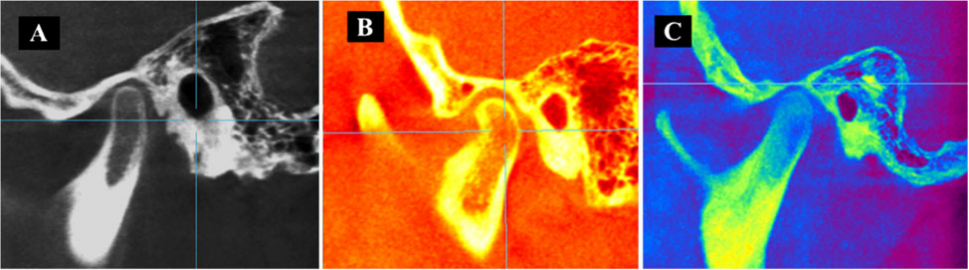
**Images of condylar position as skeletal pattern. (A)** Condylar position with respect to class I. **(B)** Condylar position in class II. **(C)** Condylar position in class III.

The spatial analysis of the condylar position was done using the anatomical landmarks proposed by Ricketts [15]. These landmarks are described in Table 2, denoting each distance as follows: upper distance (Cs-GI), posterior distance (Cp-PI), anterior distance (Ca-EI), angle of eminence (EE′.Fh′), and height of the eminence (GI-Fh′) (Figure 7). Images of the condylar position according to skeletal pattern are shown in Figure 8.

**Table 2:** **Reference points, lines, and planes used in the morphometric analysis of condyle position**

	Description
Reference points, lines, and planes	
Cs	The highest point of the condyle in the sagittal view
GI	Point of greatest concavity of the glenoid fossa
Cp	Most convex point on the posterior face of the condyle
PI	Line perpendicular to the Frankfurt plane passing through the midpoint of the sagittal diameter of the external auditory canal
Ca	Point on the anterior wall of the condyle closest to the posterior wall of the articular eminence
EI	Point on the posterior wall of the articular eminence closest to the anterior wall of the condyle
E-E′	Line tangential to the posterior wall of the articular eminence
Fh′	Line parallel to the Frankfurt plane passing through the lower edge of the articular eminence
Distances^a^	
Cs-GI	Upper distance from the highest part of the condyle to the deepest part of the glenoid fossa
Cp-PI	Posterior distance from the most convex part of the posterior wall of the condyle to line PI
Ca-EI	Anterior distance joining the most convex point on the anterior wall of the condyle with point EI
E-E′.Fh′	Angle between the tangent passing through the posterior wall of the articular eminence and the Fh′ plane parallel to the Frankfurt plane
GI-Fh	Height of the eminence from the deepest part of the glenoid fossa to the Fh′ plane parallel to the Frankfurt plane

^a^Used for measuring the superior, posterior, and anterior relationships between the condyle and the glenoid fossa.

### Statistical analysis

Statistical processing and analysis of the data was performed using SPSS statistics program for Windows (version 19.0, SPSS, Chicago, IL, USA). A reliability analysis was carried out by randomly choosing 15 images. Measurements were taken 1 week apart by two operators.

The Shapiro-Wilk normality test was applied. Analysis of variance (ANOVA), Kruskal Wallis, Mann–Whitney *U*, and Tukey's tests were finally used depending on the normality in the groups.

## Results

Intraclass intraobserver correlation coefficients of 0.92 (upper distance), 0.91 (posterior distance), 0.92 (anterior distance), 0.90 (angle of eminence), and 0.94 (height of eminence), and interobserver correlation coefficients of 0.90 (upper distance), 0.90 (posterior distance), 0.93 (anterior distance), 0.90 (angle of eminence), and 0.91 (height of eminence) were obtained.

A paired Student's *t* test showed no statistically significant differences between the two intraobserver measurements: for observer 1 (*p* = 0.374) and for observer 2 (*p* = 0.208). Also, no significant differences were identified when comparing interobserver measurements at different times (*p* = 0.178 (time 1) and *p* = 0.200 (time 2)).

Statistical description of the spatial analysis of condyle position according to skeletal pattern is given in Table 3. The upper distance from the condyle to the glenoid fossa was smaller in the class II and the class III groups compared with the class I group (*p* = 0.001 and *p* = 0.009, respectively). However, a comparison of the upper distance for the class II and class III groups revealed no significant differences (*p* > 0.763). No statistically significant differences were found for the posterior distances between the three groups (all *p* > 0.308).

**Table 3:** **Morphometric analysis of condylar position by skeletal pattern**

Skeletal pattern	Measurements	Mean	SD	Min	Max	***S*** ^2^	***p*** value	***p*** (1, 2)	***p*** (1, 3)	***p*** (2, 3)
1. Class I	Upper distance (mm)	3.02	0.58	1.90	3.80	0.34	0.001^a^	0.001^c^	0.009^c^	0.763^c^
	Posterior distance (mm)	6.70	1.00	4.80	8.20	1.02	0.311^a^	0.502^c^	0.308^c^	0.932^c^
	Anterior distance (mm)	2.44	0.50	1.70	3.60	0.25	0.080^b^	0.033^d^	0.025^d^	0.217^d^
	Angle of eminence (deg)	58.19	7.32	46.30	69.50	53.66	<0.001^b^	0.013^d^	<0.001^d^	0.004^d^
	Height of eminence (mm)	7.10	0.94	4.70	8.30	0.88	0.041^a^	0.859^c^	0.044^c^	0.134^c^
2. Class II	Upper distance (mm)	2.07	0.22	1.20	3.30	0.39				
	Posterior distance (mm)	7.25	1.58	5.10	11.10	2.51				
	Anterior distance (mm)	2.05	0.74	1.30	4.00	0.55				
	Angle of eminence (deg)	51.14	7.83	36.00	64.70	61.31				
	Height of eminence (mm)	6.89	1.15	4.80	8.70	1.32				
3. Class III	Upper distance (mm)	2.25	0.80	1.30	3.80	0.64				
	Posterior distance (mm)	7.43	1.32	5.40	9.50	1.76				
	Anterior distance (mm)	2.25	0.55	1.40	3.60	0.31				
	Angle of eminence (deg)	41.49	9.22	28.80	59.40	85.04				
	Height of eminence (mm)	6.07	1.31	9.10	9.10	1.73				

SD, standard deviation; *S* ^2^, variance. ^a^ANOVA, ^b^Kruskal-Wallis, ^c^Tukey, ^d^Mann-Whitney *U*. *p* < 0.05.

With respect to the anterior distance of the condyle to the articular eminence, it was observed that there is a statistically significant difference when comparing the class I against the class II (*p* = 0.033) and the class III (*p* = 0.025) groups. No statistically significant difference was found when comparing the class II and III groups (*p* = 0.217).

For the articular eminence angle, there was a statistically significant (*p* < 0.001) difference between the three groups, and it was further observed that the class III group showed a smaller angle of eminence than the class II group. Also, both class II and III groups had smaller angles than the class I group.

**Figure 9:**
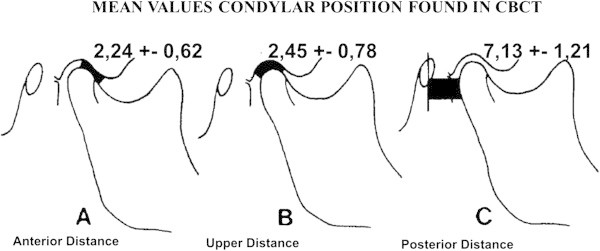
**Mean values of the condyle position found during analysis of the TMJ using CBCT images. (A)** Anterior distance. **(B)** Upper distance. **(C)** Posterior distance.

A statistically significant difference (*p* = 0.044) was identified regarding the height of the articular eminence comparing the class I with the class III group, with the class III group having a smaller value. Finally, the mean values of the condylar spatial analysis of the CBCT images (Figure 9) and the values obtained from laminography by Ricketts [15] showed no statistically significant differences, as can be seen in Table 4.

**Table 4:** **Difference in averages between Ricketts values obtained by laminography and by CBCT analysis**

Techniques	Measurements	Mean	DS	***V***_min_	***V***_max_
1. CBCT	Upper distance (mm)	2.5	0.8	1.2	3.8
	Posterior distance (mm)	7.1	1.2	4.8	11.1
	Anterior distance (mm)	2.2	0.6	1.3	4.0
	Height of eminence (mm)	6.1	1.2	3.6	8.2
	Angle of eminence (deg)	50.0	10.1	28.8	69.5
2. Laminography	Upper distance (mm)	2.5	1.0	0.5	5.5
	Posterior distance (mm)	7.5	1.5	5.0	10.0
	Anterior distance (mm)	1.5	0.5	0.5	3.0
	Height of eminence (mm)	6.3	1.3	4.0	11.5
	Angle of eminence (deg)	54.0	10.0	25.0	79.0

All *p* > 0.05, Student's *t* test.

## Discussion

Understanding the TMJ morphology and its relative position in the class I, class II, and class III groups remains a challenge for clinicians. Knowledge on the spatial variations of normal condyle-glenoid fossa relationship could allow the clinician to potentially identify the beginning of a degenerative joint disease or indicate problems already established, as well as better treatment planning where obtaining values closer to normal is indicated [14, 15]. Therefore, the accurate determination of these values in conjunction with clinical observations could be of great importance for diagnosis and treatment planning in different skeletal relationships.

Ricketts [14] used laminographic images to analyze TMJs whose images had minimum distortion. Based on this, we used CBCT images (theoretically zero distortion) [8] in order to determine the condyle spatial position in relation to the articular eminence for the different skeletal anteroposterior relationships and to compare these values with those taken from laminographic images by Ricketts [15]. In this study, we found that the condyles in the class II and class III groups are more superior than those in the class I group. Ricketts [15] and Katsavrias [12] mentioned that the condyles in class III skeletal relationship patients were closer to the roof of the glenoid fossa, which coincides with our results. Nevertheless, with regard to the class II group, Ricketts [15] stated that the condyle was positioned lower, which does not agree with our results, which may suggest that the condyle position is influenced by a variety of factors related to the included vertical pattern. The class II and class III groups selected for our study had a long vertical facial pattern. In this regard, Paredes et al. [18] mentioned that the upper distance between the condyle and fossa is increased in short facial types and reduced in long facial types, coinciding with our results. Therefore, both vertical patterns (long or short) should also be considered in determining condylar position clinically and radiographically. The clinical significance of the upper distance is that it determines whether the condyle is morphologically altered since an increase or decrease of the distance may produce pathologies such as resorption and condylar hyperplasia [14, 15].

Regarding the posterior distance, no significant differences were observed between the three groups studied. The posterior condylar position relative to a line perpendicular to the Frankfurt plane passing through the midpoint of the sagittal diameter of the external auditory canal may actually be influenced by the morphology and location of it [17]. Differences were observed indicating that the condyles of the class II and III groups were anteriorly located when compared to the class I group. Similarly, Ricketts [15] in his analysis of variations in condyle position mentions that in class II malocclusions the condyle appears to be more anterior. We suggest that future studies take into account the posterior wall of the glenoid fossa instead of the line perpendicular to the Frankfurt plane passing through the midpoint of the sagittal diameter of the external auditory canal. In our study, the parameters were determined by the criteria used by Ricketts.

With regard to the anterior distance from the condyle to the eminence, there is a statistically significant difference between the class I and the class II groups, and between the class I and class III groups, suggesting that the condyle in the class II group is positioned more anteriorly, coinciding with the results of Katsavrias [12], who stated that in class II groups of both divisions, the condyle is located in a more anterior position. Pullinger and Hollender [19] showed that a non-concentric position of the condyle is a characteristic of class II malocclusion and that the condyles are positioned further in the anterior direction in patients with class II malocclusion than in class I patients. Furthermore, our study showed that there is a statistically significant difference in the anterior distance between the class I and III groups, finding that the condyle in the class III group is in a more anterior position compared with the class I group. This result may be due to a tendency towards a non-concentric position of the condyle-glenoid fossa, morphological variability, and the degree of severity of skeletal and vertical pattern type. Rodrigues et al. [20] evaluated the concentric position in the mandibular fossa and found a non-concentric position on both right and left sides for class II and class III groups. Katsavrias [12] showed that variations in the morphology of the condyle are related principally to the inclination of the head of the condyle and that the shapes of the condyle and fossa are different in the class II and class III groups. It is important to note that if this distance decreases with respect to the normal distance and the treatment provided further decreases, this distance could face condylar distraction, highlighting the clinical importance of knowing the TMJ without alteration and according to the skeletal pattern.

With respect to the articular eminence angle, Katsavrias [21] and Sülün et al. [22] showed that a pronounced inclination in the articular eminence can predispose a dysfunctional temporomandibular joint and that class II division 2 malocclusion cases are characterized by strong and high articular eminences. However, in this investigation, the articular angle was found to be highest in the class I group (58°), followed by class II (51°), and lowest in the class III group (42°). We cannot, therefore, confirm that a pronounced inclination in the articular eminence can influence the development of the TMJ. Christiansen et al. [23] reported that the normal value of the articular eminence angle in adults is 30° to 60°, while the values measured in this study, for young adult patients, varied between 42° and 58°. Ricketts [24] says that at the age of 7.5 years the inclination is 46°, and at 12.5, 18.5, and 22 years of age, the inclination is 52°, 57°, and 59°, respectively. In this regard, we would point out that the sample used in this study had a mean age of 25 years and that the mean inclination of the eminence of the study groups was 50° with a standard deviation of ±8°, similar to the values defined by Ricketts [24]. The ratio of the inclination of the articular eminence within different skeletal pattern types can be affected by other factors related to age, sex, dental occlusion, and incisor or canine guidance, among other factors. Most of these variables were not considered in our study.

The articular eminence height was found to be lower in the class III group when compared with the class I group. This result could be due to the influence of facial vertical pattern length and severity of the skeletal relationship. Regarding this, Katsavrias [12] mentions that variations in the shape of the fossa are related to the inclination and height of the eminence and stated that in the class III group the condyle is higher or closer to the roof of the fossa. Vitral et al. [25] did not find any significant differences in the height of the glenoid fossa between the class I and class II groups, results that coincide with those obtained in this study. Moreover, Ricketts [15], Cohlmia et al. [26], and Vitral et al. [25] found no correlation between the depth of the glenoid fossa, the inclination of the eminence, and the different dental malocclusions.

The values of the spatial analysis of condylar position in CBCT images of the three study groups, class I, class II, and class III (Figure 9), are within the ranges shown by Ricketts in his study of condylar position in laminographic images. These similarities maybe due to the low image distortion in laminography and the relative absence of distortion in CBCTs. It has to be noted that the measurements obtained in this study should be more accurate because the CBCT images are isotropic.

### Limitations

The attempt of this initial study was to evaluate the precision of TMJ internal linear and angular measurements as measured with CBCT images. An important limitation regarding temporomandibular dysfunction (TMD) diagnosis was identified. In this study, it was not possible to measure the daily and chronic stress of patients, habits, pathological and functional changes, and muscle activity that have been shown to impact TMD.

Although CBCT maybe one useful tool for measuring TMJ bony structures given the improved anatomical resolution it provides, one should not forget the ALARA principles when determining the need for further images and related increased ionizing radiation. Careful consideration of a cost/benefit analysis should be encouraged. Also, it should be noted that although the results obtained in this study show statistically significant differences, the clinical relevance of the differences is questionable.

## Conclusions

From the study, the following points were found:

1. Only some spatial differences in condylar position inside the glenoid fossa were identified between skeletal class I, class II, and class III malocclusions.
2. Class II and class III malocclusion condyles with vertical long pattern are located more anteriorly and superiorly than class I malocclusions.
3. Class III malocclusion articular eminence height is smaller than in class I malocclusions.
4. There are significant differences in articular eminence angle among the three malocclusion groups.

## Abbreviations

CBCT: Cone beam computed tomography
ANOVA: Analysis of variance
TMD: Temporomandibular dysfunction
TMJ: Temporomandibular joint
3D: Three-dimensional
MIP: Multiplane image.
